# miR-145-5p and miR-148b-3p Expression Is Inversely Associated with Pten Expression in Prostate Pathologies

**DOI:** 10.3390/cimb47090782

**Published:** 2025-09-22

**Authors:** Karla Lizbeth Morales Hernández, Noemí García Magallanes, Marco Alvarez Arrazola, Fred Luque Ortega, Martín Irigoyen Arredondo, Fernando Bergez Hernandez, Eliakym Arambula Meraz

**Affiliations:** 1 Posgrado en Ciencias Biomédicas, Facultad de Ciencias Químico Biológicas, Universidad Autónoma de Sinaloa, Culiacán 80010, Mexico; karla.morales@uadeo.mx; 2 Licenciatura en Ciencias Biomédicas, Universidad Autónoma de Occidente, Mazatlán 82100, Mexicofernando.bergez@uadeo.mx (F.B.H.); 3 Laboratorio de Biomedicina y Biología Molecular, Ingeniería en Biotecnologia, Universidad Politécnica de Sinaloa, Mazatlán 82199, Mexico; ngarcia@upsin.edu.mx (N.G.M.);; 4 Alvarez & Arrazola Radiólogos, Mazatlán 82140, Mexico; 5 Laboratorio de Ciencias Básicas, Facultad de Odontología, Universidad Autónoma de Sinaloa, Culiacán 80013, Mexico; 6 Laboratorio de Genética y Biología Molecular, Facultad de Ciencias Químico Biológicas, Universidad Autónoma de Sinaloa, Culiacán 80010, Mexico

**Keywords:** prostate cancer, miRNAs, PTEN, correlation

## Abstract

Prostate cancer (PCa) represents a significant cause of cancer-associated mortality in the male population worldwide and constitutes a multifactorial disease influenced by genetic and epigenetic factors. Deregulation of genes such as AR and PTEN, as well as alteration in the expression of microRNAs (miRNAs), including miR-145-5p and miR-148b-3p, has been observed in this pathology. This study aimed to explore the correlation between the expression of miR-145-5p, miR-148b-3p, and PTEN in prostate tissue, providing initial insight into their potential interaction in cancer biology. We analyzed 71 samples, comprising 41 from patients with confirmed prostate cancer (PCa group) and 30 from patients with benign prostatic disease (BPD group). Our findings demonstrated a statistically significant association between both miRNAs and the PTEN gene, specifically between miR-148b-3p and PTEN (*p* = 0.00001) and between miR-145-5p and PTEN (*p* = 0.0078). These findings support the hypothesis that reduced levels of these miRNAs may be linked to PTEN regulation in prostate pathologies and underscore their potential relevance in PCa biology.

## 1. Introduction

Prostate cancer (PCa) is the second most frequent malignancy in men worldwide, with 1,467,854 new cases and 397,430 PCa-related deaths in 2022, according to the International Agency for Research on Cancer (IARC), World Health Organization. Although prostate-specific antigen (PSA) has contributed to earlier detection, it is also associated with high false-positive rates, overdiagnosis, and overtreatment, particularly in developed countries [1,2].

The etiology of PCa is multifactorial and involves the interaction of various genes in its development and progression [3]. One of the most critical molecular drivers is the androgen receptor (AR), a gene located on the X chromosome that encodes a protein of 919 amino acids. This AR protein consists of three functional domains encoded by eight exons [4]. In its inactive state, AR resides in the cytoplasm bound to chaperone proteins. Upon activation by androgenic hormones—testosterone or dihydrotestosterone (DHT), with the latter being the more potent ligand—AR dissociates from its chaperones and forms a complex with its ligand. In the cytoplasm, testosterone is metabolized to DHT via the enzyme 5α-reductase. The androgen–AR complex then translocates into the nucleus, where it dimerizes and binds to androgen response elements (AREs) within the regulatory regions of target genes, thereby influencing their transcription. These targets commonly include genes that regulate the cell cycle, proliferation, and other essential processes required for normal prostate development and function [5,6]. However, in PCa, these events do not occur normally and are instead affected by modifications in AR, such as splice variants, mutations and gene amplifications, leading to cancer progression and resistance to androgen deprivation therapy in patients. Prostate growth regulated by androgens is crucial in the pathology’s progression, as it supports not only the prostate’s general function but also the growth and survival of both healthy and cancerous prostate tissues [5].

The phosphatase and tensin homolog (PTEN) gene, mapped to chromosome 10q23.31, functions as a crucial regulator of cellular processes such as proliferation, motility, and survival. PTEN is widely recognized as a major tumor suppressor, and its somatic alterations, such as point mutations, deletions, or epigenetic silencing via hypermethylation, can lead to the loss or inactivation of one allele, a characteristic observed in several advanced cancers, including PCa. To maintain cellular homeostasis, the PTEN protein product acts as an antagonist of phosphatidylinositol 3-kinase (PI3K), dephosphorylating its substrate PIP3 to PIP2, leading to a negative regulation of the PI3K/AKT pathway. The regulation of PTEN can be affected at the post-transcriptional level by microRNAs [7,8,9].

miRNAs are small, single-stranded, non-coding RNAs of approximately 18–25 nucleotides in length. Their primary function is to regulate gene expression at the post-transcriptional level, typically through binding to complementary sequences in the 3′ untranslated regions (3′UTRs) of target messenger RNAs [9]. Depending on the cellular context and the specific targets that they regulate, miRNAs may exert oncogenic activity (oncomirs) or tumor-suppressive functions (oncosuppressors). Aberrant miRNA expression has been associated with the initiation and progression of various types of cancer, including PCa. Notably, significant differences in miRNA expression profiles have been observed between normal and neoplastic prostate tissue, supporting their functional relevance in the development of tumorigenesis [7].

Approximately one-third of the human genome is under the regulatory control of miRNAs, whose abnormal expression profiles are strongly associated with cancer progression. Generally, miRNAs repress gene expression by attaching to complementary sequences within the 3′UTR of target genes; nevertheless, binding events at the 5′UTR have also been reported, albeit less commonly [10].

miR-145-5p, which is part of the miR-145 family, has been identified at the chromosomal locus 5q33.1. Aberrant expression of this microRNA has been observed in PCa, where it is generally considered a tumor suppressor [11]. Evidence from multiple studies indicates that miR-145-5p is downregulated in PCa tissue compared to in normal tissue, suggesting that this alteration may contribute to the pathogenesis of PCa [12].

Dysregulation of miR-148b-3p expression has been reported in several cancer types, including renal and gastric cancers and osteosarcoma, and its downregulation has been associated with poor disease prognosis [13]. Accordingly, this study sought to investigate the expression patterns of miR-145-5p and miR-148b-3p, examine their potential regulatory interactions with the AR and PTEN genes in prostate pathologies, and explore their possible involvement in PCa biology.

## 2. Materials and Methods

### 2.1. Patient Samples

Prostate tissue samples were obtained through a transrectal ultrasound-guided biopsy performed by a specialist physician. Immediately after collection, samples were preserved in QIAzol Lysis Reagent (Qiagen, Hilden, Germany) and stored at −20 °C until RNA extraction. A total of 71 samples were analyzed, comprising 41 from patients with histologically confirmed prostate cancer (PCa group) and 30 from patients with benign prostatic disease (BPD group).

Eligibility criteria included Mexican men aged 18 years or older with a confirmed histopathological diagnosis of PCa or BPD, who had not received chemotherapy or radiotherapy prior to enrollment. Samples were obtained at the Alvarez & Arrazola Radiologists Clinic in Sinaloa, Mexico, between 2016 and 2021. Clinical and pathological information—such as age, weight, height, body mass index (BMI), PSA concentration, and family history of cancer—was collected through structured questionnaires and institutional medical records. Histological assessment and Gleason scoring were performed by an experienced pathologist. Written informed consent was obtained from all participants, and the study protocol received approval from the institutional Ethics and Research Committee.

### 2.2. RNA Extraction and RT-PCR

Prostate tissue samples were employed for RNA extraction. Total RNA, including microRNAs, was purified using an miR-NEasy kit (Qiagen, Hilden, Germany) following the manufacturer’s guidelines. RNA concentration and integrity were assessed with a GENESYS 10S UV–Vis spectrophotometer (Thermo Scientific™, Waltham, MA, USA), and each measurement was performed in triplicate. For RT-PCR, 10 ng of total RNA was reverse-transcribed using a TaqMan Advanced miRNA cDNA Synthesis Kit (Applied Biosystems, Foster City, CA, USA) on a T100 thermal cycler (Bio-Rad, Hercules, CA, USA).

### 2.3. Relative Expression

Quantitative real-time PCR (qPCR) was carried out on a StepOnePlus™ thermal cycler (Applied Biosystems, Foster City, CA, USA). For each miRNA, five-point standard curves prepared from 1:10 serial dilutions were used to calculate amplification efficiency from the slope. Expression levels of miRNAs (miR-145-5p and miR-148b-3p) and genes (AR and PTEN) were quantified with TaqMan MicroRNA Assays (Applied Biosystems, Foster City, CA, USA). miR-191-5p served as the reference control for miRNAs, while GAPDH was used as the reference gene. Relative quantification was determined following the approach of Taylor et al. [14], which is based on the ∆∆Ct method (2^−∆∆Ct^ algorithm) described by Livak and Schmittgen [15]. Log-transformed normalized relative expression values were subsequently used for statistical analysis.

### 2.4. Statistical Analysis

Descriptive statistics were obtained for all variables. Quantitative data are summarized as means ± standard deviations, and categorical variables are summarized as frequencies and percentages. Comparisons between the PCa and BPD groups were carried out using the Mann–Whitney U test. Correlation analyses were performed with Spearman’s rank correlation coefficient, depending on the data distribution. These tests were applied to examine the associations between the expression levels of miRNAs and genes (miR-145-5p, miR-148b-3p, PTEN, and AR). Statistical analyses were conducted with IBM SPSS Statistics version 20 (IBM Corp., Armonk, NY, USA), and results with *p* < 0.05 were considered statistically significant.

## 3. Results

### 3.1. Clinicopathological Characteristics

The mean age of the patients in the PCa group was significantly greater (66.5 years) than that of the patients in the BPD group (62.4 years, *p* < 0.01 **). No significant differences were detected between the groups in terms of BMI or PSA levels. The distribution of Gleason scores in the PCa group showed that most patients had a score of 7 (63.4%), with similar proportions of 3 + 4 (ISUP grade 2) and 4 + 3 (ISUP grade 3). Scores ≥ 9 were observed in 22% of patients, while only 4.9% had a score ≤ 6 (ISUP grade 1) (Table 1).

### 3.2. Expression Analysis

Analysis of relative gene expression revealed that AR was underexpressed in PCa samples, with a fold change of 0.75 compared to the BPD group. However, this difference was not statistically significant (*p* = 0.084). Similarly, PTEN expression showed a fold change of 1.08 in PCa tissues relative to BPD tissues, with no significant difference observed (*p* = 0.572).

**Figure 1:**
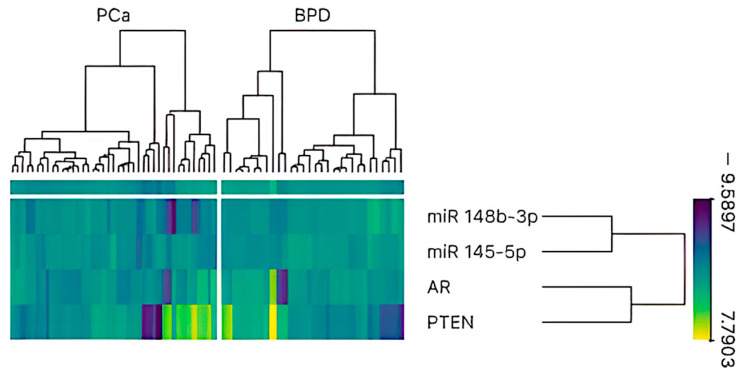
Heatmap and hierarchical clustering analysis of miR-145-5p, miR-148b-3p, AR, and PTEN expression in prostate tissue samples (*n* = 71). Columns represent individual samples categorized into two groups: prostate cancer (PCa) and benign prostate disease (BPD). Rows correspond to normalized expression levels of the selected genes and miRNAs. Color scale indicates expression intensity, with yellow representing high expression and purple indicating low expression. Hierarchical clustering reveals distinct expression patterns between PCa and BPD groups, with a tendency toward reduced expression of miR-145-5p and miR-148b-3p and increased expression of AR and PTEN in PCa samples.

As previously reported by our research group, both miR-145-5p and miR-148b-3p were significantly underexpressed in PCa samples compared to BPD samples. Specifically, miR-145-5p showed a fold change of 0.71 (*p* < 0.05 *), while miR-148b-3p exhibited a more pronounced reduction, with a fold change of 0.44 (*p* < 0.001 ***) (Figure 1) [16].

### 3.3. Correlation Analysis

**Figure 2:**
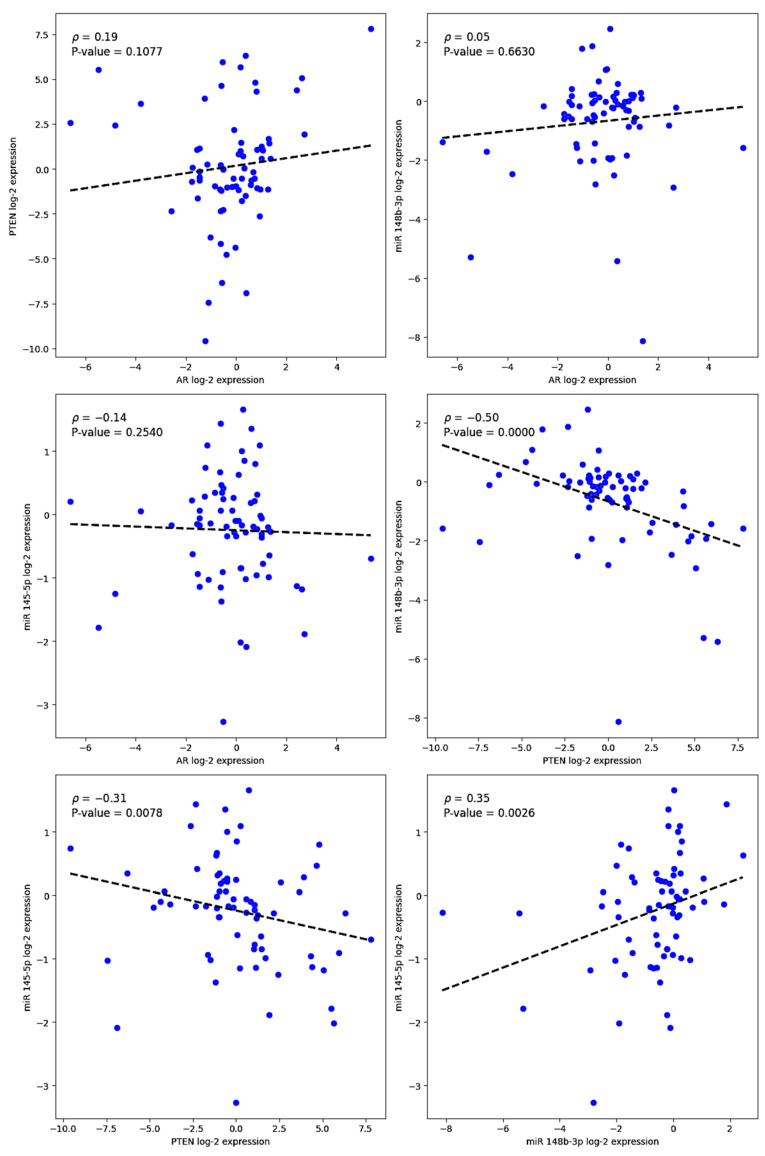
Scatter plots showing Spearman correlation analyses between log_2_-transformed expression levels of miR-145-5p and miR-148b-3p and those of AR and PTEN in prostate tissue samples (*n* = 71). Each dot represents an individual sample. Dashed lines indicate linear regression trends for visualization. Statistically significant inverse correlations were observed between PTEN and both miR-145-5p (ρ = −0.31, *p* = 0.0078) and miR-148b-3p (ρ = −0.35, *p* = 0.0026). No significant correlations were found between either of these miRNAs and AR expression.

Correlation analyses were performed to evaluate the relationships between gene and miRNA expression levels and various clinicopathological parameters (Table 2). Notably, a statistically significant inverse correlation was observed between miR-148b-3p and PTEN (Spearman’s r = −0.53, *p* < 0.001 ***), as well as between miR-145-5p and PTEN (Spearman’s r = −0.35, *p* < 0.01 **) (Figure 2). No significant correlations were found between the expression of either of these miRNAs and AR.

## 4. Discussion

In this study, correlation analyses revealed a statistically significant inverse association between PTEN expression and both miR-145-5p and miR-148b-3p. In contrast, no significant correlations were observed between either of these miRNAs and AR gene expression. Although AR is recognized as one of the most miRNA-regulated oncogenes in PCa and plays a pivotal role in prostate growth and disease progression, our findings did not demonstrate a direct correlation with the miRNAs analyzed. The AR signaling axis is known to be modulated at multiple levels by a variety of regulatory elements, including corepressors, coactivators, transcription factors, chaperones, long non-coding RNAs (lncRNAs), and miRNAs [6].

AR is characterized by an unusually long 3′ untranslated region (3′UTR), approximately 4 kb in length, enriched with AU-rich elements. This structure renders AR particularly susceptible to post-transcriptional regulation by miRNAs, similar to other members of the steroid hormone receptor family. Several mechanisms have been described through which AR and miRNAs interact. First, AR can directly bind to the promoter regions of miRNA genes, modulating their transcription. Second, AR can regulate miRNA expression indirectly via AR-responsive transcription factors that also target miRNA promoters. Third, AR-driven RNA-binding proteins may influence miRNA stability post-transcriptionally. Moreover, certain miRNAs participate in feedback regulatory loops with AR, collectively contributing to the fine-tuning of androgen signaling pathways [10].

The feedback loop between miRNAs and their target genes can be either positive or negative. The extent of sequence complementarity and the number of binding sites between an miRNA and its target mRNA influence the outcome—ranging from mRNA degradation to translational repression—thereby determining the biological effect. Importantly, miRNA-mediated regulation is not a linear process but rather part of a highly interconnected regulatory network. For example, androgens that upregulate AR expression also promote the expression of AR-repressive miRNAs such as miR-31 and miR-135a, which frequently engage in mutual repression with AR [6]. The lack of a direct correlation between AR and the miRNAs analyzed in this study may reflect the complexity of such regulatory mechanisms, potentially involving post-transcriptional repression, indirect interactions, or multifactorial feedback circuits.

**Figure 3:**
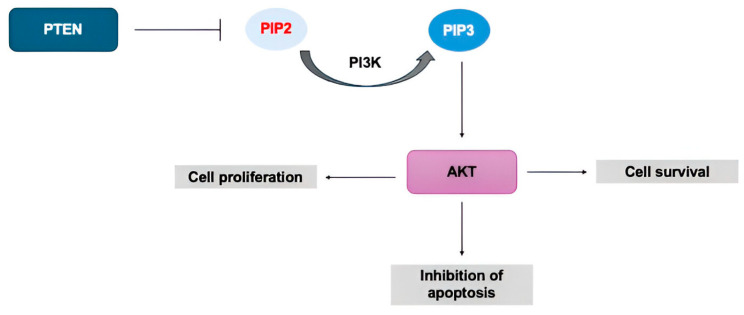
PTEN-PI3K-AKT pathway. PTEN suppresses the phosphorylation of PIP2 to PIP3, inhibiting the activation of AKT, which is involved in processes such as cell survival, proliferation, and inhibition of apoptosis.

The tumor suppressor PTEN and the PI3K signaling pathway that it regulates are among the most frequently altered molecular axes in primary prostate cancer [17]. PTEN functions as a critical negative regulator of the PI3K/AKT pathway. Through its lipid phosphatase activity, PTEN converts phosphatidylinositol (3,4,5)-trisphosphate (PIP3) into phosphatidylinositol (4,5)-bisphosphate (PIP2), thereby attenuating AKT activation and maintaining normal cell cycle control. Loss or underexpression of PTEN leads to PIP3 accumulation, resulting in sustained activation of the PI3K/AKT pathway and promoting uncontrolled cellular proliferation (Figure 3) [3,17].

**Figure 4:**
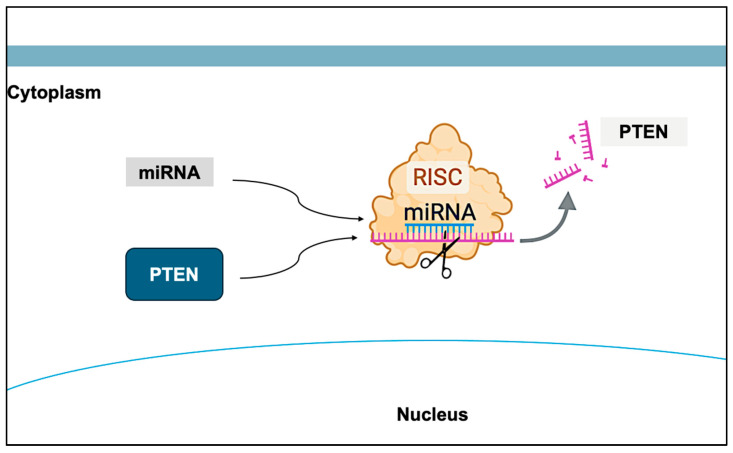
Effect of miRNAs on PTEN. The expression of PTEN can be influenced by epigenetic factors, such as miRNAs, which act as post-transcriptional regulators of PTEN. Created with BioRender.com.

PTEN expression can be modulated by various epigenetic mechanisms, including DNA methylation, histone modifications, and miRNAs, which act as post-transcriptional repressors of this tumor suppressor gene (Figure 4) [7,8,18]. The expression of these regulatory elements may be influenced by genomic events such as chromosomal rearrangements, deletions, amplifications, mutations, and promoter hypermethylation [19]. A wide range of miRNAs—including miR-21, miR-126, miR-132, miR-200, miR-214, miR-382-5p, and miR-486-5p—have been shown to target key nodes within the PI3K/AKT/mTOR signaling cascade. These miRNAs have been implicated in the pathogenesis of numerous malignancies, including promyelocytic leukemia, esophageal squamous cell carcinoma, renal cell carcinoma, breast cancer, multiple myeloma, osteosarcoma, endometrial cancer, glioma, lung cancer, colorectal cancer, and PCa [18].

miR-145-5p has been experimentally validated as a direct post-transcriptional regulator of the tumor suppressor gene PTEN, as reported in miRTarBase, a curated database of microRNA–target interactions. Functional assays, including luciferase reporter analyses and gene expression profiling, have confirmed that miR-145-5p binds to the 3′ untranslated region (3′UTR) of PTEN mRNA, leading to its downregulation [20]. Computational predictions from TargetScan further support this interaction, identifying a conserved binding site for miR-145-5p in the PTEN 3′UTR. The associated Context++ score is approximately −0.40, which denotes a high likelihood of effective silencing. This score integrates various features, including seed match quality, evolutionary conservation, and site accessibility, offering a robust prediction of miRNA–mRNA interaction efficiency [21].

According to TargetScan predictions, the PTEN gene harbors putative binding sites within its 3′UTR for members of the miR-148/152 family, which includes miR-148b-3p. Given that these miRNAs share an identical seed sequence, it is highly probable that miR-148b-3p binds to the PTEN 3′UTR, exerting post-transcriptional repression. This potential interaction suggests a functional role for miR-148b-3p in modulating PTEN expression, thereby impacting key biological processes regulated by PTEN, including cell proliferation control, apoptosis, and tumor suppression [21].

Recently, there has been increasing interest in elucidating the role of microRNAs in PTEN regulation, particularly in the context of PCa. Notably, studies such as that of Tian et al. [3] have demonstrated that miRNAs, including miR-19b, miR-23b, miR-26a, and miR-92a, can promote prostate cell proliferation by modulating PTEN expression and activating downstream signaling pathways. In another study, researchers assessed the expression levels of miR-let-7b-3p and miR-548c-3p in tissue samples from PCa, BPD, and adjacent normal prostate tissue, concluding that overexpression of miR-548c-3p in PCa may contribute to PTEN downregulation [19]. Furthermore, a separate investigation reported an inverse association between miR-106a and PTEN expression in both PCa tissues and PC-3 cell lines, confirming that miR-106a directly targets PTEN in vitro, thereby potentially participating in tumorigenic signaling networks [22].

In a comprehensive 2023 analysis, Selvakumar reviewed multiple studies involving PCa patients and cell lines, concluding that the aberrant expression of miR-92a, miR-181a-5p, and miR-186-5p may contribute to PCa development through modulation of the PTEN/PI3K/AKT signaling pathway. Dysregulation of this axis can promote key tumorigenic processes, including cell proliferation, migration, and invasion, thereby highlighting the pivotal role of miRNAs in PCa progression [8].

In our study, we identified a negative correlation between both miRNAs and PTEN, suggesting that decreased expression of miR-145-5p and miR-148b-3p is associated with increased PTEN expression. The established role of miR-145-5p as a tumor suppressor highlights its therapeutic potential, as restoring its expression may help inhibit tumor growth and prevent metastatic progression in PCa. Similarly, the consistent underexpression of miR-148b-3p in oncological contexts suggests that it may also function as a tumor suppressor, contributing to the repression of oncogenic pathways. In PCa, reduced levels of miR-148b-3p could facilitate tumor progression by impairing mechanisms that typically constrain malignant cell proliferation and dissemination [7,23].

Although PTEN functions as a tumor suppressor, and its increased expression is generally associated with the inhibition of cell proliferation via the PI3K/AKT signaling pathway, its regulation may not follow a linear or predictable pattern in all oncogenic contexts. One possible explanation for these discrepancies is that, in some cases, elevated PTEN expression may be insufficient to counteract concurrent molecular alterations, such as mutations in other signaling components or the influence of additional miRNAs and epigenetic modifiers. In the case of miR-145-5p and miR-148b-3p, their downregulation may lead to impaired regulation of PTEN, indirectly favoring the activation of proliferative pathways and contributing to tumor progression. Thus, despite PTEN’s critical role in tumor suppression, its biological impact is context-dependent and influenced by the complexity of the tumor microenvironment and the broader regulatory network. Therefore, future investigations incorporating functional validation will be crucial to elucidate the underlying molecular mechanisms and to confirm the interactions predicted by databases such as miRTarBase and TargetScan.

## 5. Conclusions

Understanding the interaction between PTEN and miRNAs is essential, as it provides insight into the molecular mechanisms underlying the transition from normal to malignant prostate tissue. Our findings revealed a notable negative association between PTEN expression and the expression of miR-145-5p and miR-148b-3p, suggesting a potential regulatory relationship. This statistical correlation points to a possible epigenetic mechanism involved in PCa biology and opens new avenues with the goal of developing targeted therapeutic interventions to modulate miRNA expression and their interactions with key tumor suppressor genes.

## Figures and Tables

**Table 1:** Summary of relevant clinical parameters.

Clinicopathological Characteristics	Group	Average/Distribution	Statistical Significance
Age	PCa	66.55 ± 12.13 years	*p* < 0.01 **
	BPD	62.45 ± 6.93 years	
BMI	PCa	24.67 ± 2.77 kg/m^2^	*p* = 0.23
	BPD	24.36 ± 5.52 kg/m^2^	
PSA	PCa	65.91 ± 203 ng/mL	*p* = 0.774
	BPD	71.85 ± 178.26 ng/mL	
Gleason score	PCa	≤6: 4.9% (ISUP 1) 7 (3 + 4): 31.7% (ISUP 2) 7 (4 + 3): 31.7% (ISUP 3) 8: 9.8% (ISUP 4) ≥9: 22.0% (ISUP 5)	-

Mann–Whitney U test. Statistical significance is indicated as follows: ** *p* < 0.01.

**Table 2:** Correlation between relative expression of genes and clinicopathological characteristics.

Variable	Statistics Values	AR	PTEN	miR 145-5P	miR 148B-3P
PSA	correlation coefficient	0.032	−0.110	−0.051	−0.217
	*p*-value	0.846	0.500	0.755	0.179
Age	correlation coefficient	−0.004	−0.133	−0.121	0.066
	*p*-value	0.971	0.283	0.331	0.598
Weight	correlation coefficient	0.041	−0.155	−0.128	0.326
	*p*-value	0.837	0.432	0.518	0.091
BMI	correlation coefficient	−0.200	−0.239	0.043	0.167
	*p*-value	0.317	0.230	0.831	0.404

Spearman’s correlation coefficients (ρ) are shown. PSA: prostate-specific antigen. BMI: body mass index.

## Data Availability

The data presented in this study are available on request from the corresponding author.

## Abbreviations

The following abbreviations are used in this manuscript:

PCa	Prostate Cancer
PSA	Prostate-Specific Antigen
AR	Androgen Receptor
DHT	Dihydrotestosterone
PTEN	Phosphatase and Tensin Homolog
PI3K	Phosphatidylinositol 3-Kinase
PIP3	Phosphatidylinositol (3,4,5)-Triphosphate
PIP2	Phosphatidylinositol (4,5)-Bisphosphate
AKT	Protein Kinase B
miRNA	MicroRNA
BPD	Benign Prostatic Disease
mTOR	Mechanistic Target of Rapamycin
qPCR	Quantitative Polymerase Chain Reaction
GAPDH	Glyceraldehyde-3-Phosphate Dehydrogenase
ΔΔCt	Delta Delta Cycle Threshold
